# Tedizolid, Faropenem, and Moxifloxacin Combination With Potential Activity Against Nonreplicating *Mycobacterium tuberculosis*

**DOI:** 10.3389/fphar.2020.616294

**Published:** 2021-01-19

**Authors:** Shashikant Srivastava, Kayle N. Cirrincione, Devyani Deshpande, Tawanda Gumbo

**Affiliations:** ^1^ Center for Infectious Diseases Research and Experimental Therapeutics, Baylor Research Institute, Baylor University Medical Center, Dallas, TX, United States; ^2^ Department of Immunology, UT Southwestern Medical Center, Dallas, TX, United States; ^3^ Department of Pulmonary Immunology, University of Texas Health Science Center at Tyler, Tyler, TX, United States; ^4^ Praedicare Laboratories and Quantitative Preclinical & Clinical Sciences Department Praedicare Inc., Dallas, TX, United States; ^5^ Department of Medicine, University of Cape Town, Observatory, Cape Town, South Africa

**Keywords:** special population, tuberculosis, nonreplicating persisters, regimen, hollow fiber model

## Abstract

**Background:** *Mycobacterium* *tuberculosis* [*Mtb*] could be present in different metabolic population in the lung lesions, and nonreplicating persisters [NRP], associated with latent tuberculosis [TB], are the most difficult to kill.

**Objective:** Test the combination of tedizolid, moxifloxacin, and faropenem for activity against NRP using *Mtb* SS18b in the hollow fiber model [HFS-TB].

**Methods:** Tedizolid and moxifloxacin were tested as, first, two-drug combination against log-phase growth [LPG] and, second, slowly replicating bacilli [SRB] under acidic condition and with faropenem to create a three-drug combination regimen. Finally, standard regimen [isoniazid-rifampin-pyrazinamide] was used as comparator in the HFS-TB experiment with NRP *Mtb*. HFS-TB units were sampled for drug-concentration measurement as well as for estimation of bacterial burden using solid agar and mycobacterial growth indicator tube [MGIT] method. Linear regression was used to calculate the kill slopes with each treatment regimen and analysis of variance (ANOVA) to compare the regimen.

**Results:** Tedizolid at standard dose in combination with high-dose moxifloxacin killed 3.05 log_10_ CFU/ml LPG *Mtb* and 7.37 log_10_ CFU/ml SRB in the bactericidal and sterilizing activity HFS-TB experiments, respectively. There was no statistical difference between tedizolid-moxifloxacin-faropenem combination and the standard regimen as both killed 7.35 log_10_ CFU/ml NRP *Mtb* in 21 days. There was no emergence of resistance to any of the drugs studied in the three HFS-TB experiments.

**Conclusion:** The experimental regimen of tedizolid, moxifloxacin, and faropenem could effectively kill NRP population of *Mtb*, and given the efficacy against different metabolic population of *Mtb* could serve as a pan-TB regimen. Clinical studies are warranted to validate the *in vitro* findings.

## Introduction

Explaining the basic mechanism of antituberculosis chemotherapy, Prof. Denis Mitchison in 1979 proposed the special population hypothesis, where each drug in the combination therapy had efficacy against a specific metabolic population of *Mycobacterium tuberculosis* [*Mtb*] (Mitchison, 1979). Isoniazid was suggested to be most active against the actively replicating bacilli, rifampin, and pyrazinamide having sterilizing activity against bacteria growing under acidic condition, and lack of drugs to have activity against dormant bacilli [nonreplicating persisters, NRP] (Mitchison, 1979). However, Mandal et al. (2019) recently published a review summarizing the problem of persisters in tuberculosis and the difficulties they posed to the tuberculosis drug discovery as well as potential role of pyrazinamide against this metabolic population. It is estimated that nearly one-third of the world’s population is latently infected. *Mtb* can enter a dormant or latent state that is characterized by limited growth and metabolism and, consistent with Prof. Mitchison’s hypothesis, this metabolic state displays phenotypic resistance to many of the commonly used drugs for treating tuberculosis [TB] (Chan and Flynn, 2004; Boshoff et al., 2005). This allows *Mtb* to persist indefinitely in the human host.

In the recent years, progress has been made to develop new models to study the efficacy of the drugs against NRP *Mtb*. One such tool is streptomycin-starved *Mtb* 18b [SS18b] strain that grows only when streptomycin is present in the growth medium (Zhang et al., 2012). Using the SS18b strain, Zhang et al. tested *in vitro* killing efficacy of 22 drugs against this special *Mtb* metabolic population and found that some of the first-line antituberculosis drugs [rifamycins and pyrazinamide] as well as new/repurposed drugs [e.g., tedizolid] have efficacy against SS18b, hence against latent TB (Zhang et al., 2012). Another recent review article summarized the killing efficacy of the above-mentioned drugs as well as that of moxifloxacin, isoniazid, and carbapenems alone or in combination of a beta-lactamase inhibitor (Iacobino et al., 2017). There was a dose-dependent killing of NRP with these drugs.

In order to access the extent of kill of NRP with three of the above-mentioned antibiotics, namely, tedizolid, moxifloxacin, and faropenem, that we found to be effective against log-phase growth [LPG] and semidormant bacilli [SRB] growing under acidic condition as well as against intracellular *M. tuberculosis*, we performed pharmacokinetics/pharmacodynamics studies using the hollow fiber model system of tuberculosis [HFS-TB] (Gumbo et al., 2009; Srivastava and Gumbo, 2011; Deshpande et al., 2016a; Swaminathan et al., 2016; Srivastava et al., 2018). HFS-TB is a preclinical drug development tool that can mimic human-like concentration-profile of the drugs and different half-life of drugs in the same systems while testing combination therapy regimens, approved by the European Medicines Agency, editorially endorsed by the United States FDA and have a predictive accuracy of over 94% (Chilukuri et al., 2015; European-Medicines-Agencies, 2015; Gumbo et al., 2015). Regarding the mechanism of action of the drugs we used in the current study, tedizolid works via inhibition of bacterial protein synthesis by binding to 23S ribosomal RNA (rRNA) of the 50S subunit of the ribosome (Zhanel et al., 2015), moxifloxacin's bactericidal action occurs via preventing replication, transcription, and repair of bacterial DNA by binding to the topoisomerase enzymes II (DNA gyrase) (Ginsburg et al., 2003), and faropenem, that has strong affinity for the high molecular penicillin binding proteins of the bacteria cell wall, inhibits transpeptidation that results in inhibition of cell wall biosynthesis of the bacteria (Gettig et al., 2008). Therefore, combining these three drugs with different mechanism of action as well as potential synergy could create an effective treatment regimen with minimal likelihood of developing cross-resistance.

## Materials and Methods

### Bacteria, Drug, and Supplies

All experiments were performed in a biosafety level 3 laboratory following the protocols approved by the Baylor Research Institute Biosafety Committee. We used *Mtb* H37Rv (ATCC27294) in the bactericidal activity and sterilizing activity experiments at acidic pH of 5.8 with detailed method as published elsewhere (Gumbo et al., 2009). *Mtb* SS18b strain (streptomycin auxotroph) was used in the NRP HFS-TB study. Streptomycin at 50 mg/L concentration was added to the growth medium of *Mtb* SS18b strain, a prerequisite growth condition as described earlier (Zhang et al., 2012). Tedizolid (the active moiety of the prodrug tedizolid phosphate) and faropenem were purchased from BOC Sciences (Shirley, NY), moxifloxacin from Baylor Medical Center Pharmacy, and isoniazid, rifampin, and pyrazinamide were purchased from Sigma Aldrich (St. Louis, MO). Hollow fiber cartridges were purchased from FiberCell Systems Inc. (Frederick, MD). Becton Dickinson (Franklin Lakes, NJ) was used to purchase the mycobacterial growth tube indicator system (BACTEC™ MGIT™ 960) and related supplies.

### Tedizolid, Moxifloxacin, and Faropenem as Two- and Three-Drug Combination Compared to the Standard Therapy

Before performing the HFS-TB experiments, MIC of the moxifloxacin, tedizolid, and faropenem [concentration range 0, 0.06, 0.12, 0.25, 0.5, 1, 2, 4, and 8 mg/L] was determined using the broth microdilution method (CLSI, 2018). We performed three different sets of hollow fiber experiments with three different *M. tuberculosis* metabolic populations, namely, actively replicating log-phase growth [LPG] bacilli at normal pH, slowly replicating bacteria [SRB] under acidic pH of 5.8, and nonreplicating persisters [NRP]. The details of the HFS-TB and methods to transform the LPG to SRB cultures have been published previously and were used without any modifications (Gumbo et al., 2009; Srivastava and Gumbo, 2011).

In the first HFS-TB study, two-drug combination of tedizolid and moxifloxacin was tested for bactericidal activity using 20 ml of LPG cultures inoculated into the peripheral compartment of each HFS-TB and treated with combination of tedizolid at standard dose of 200 mg and moxifloxacin high dose of 800 mg daily for 21 days. We mimicked a 12-h half-life for both tedizolid and moxifloxacin (Deshpande et al., 2016a; Srivastava et al., 2018). The central compartments of the HFS-TB receiving drug treatment were sampled at 0, 1, 4, 6, 10, 18, 21, and 23.5 h after starting the drug treatment to measure the drug concentration for validation of the intended concentration-time profile of the drugs. The peripheral compartments were sampled on days 0, 7, 10, 14, and 21. The samples were washed twice to remove carry-over drug, serially 10-fold diluted, and 200 μL of the processed sample was inoculated on Middlebrook 7H10 agar supplemented with 10% oleic acid-albumin-dextrose-catalase (OADC) (herein termed “agar”) to enumerate the total bacterial burden. The plates were incubated at 37°C in incubators with 5% CO_2_ for 21 days after which the colony forming units (CFU) were recorded. One portion of the sample was also inoculated into the MGIT tubes and time to positive (TTP) was recorded to confirm the growth of *Mtb*. We set the incubation period of the MGIT tubes or the “time in protocol” to 56 days in order to capture the relapse of failure of the regimens (Deshpande et al., 2016b). To capture the drug resistant subpopulation, the same samples were cultured on agar supplemented with 3X MIC of the respective drugs. The cultures were incubated for 28 days before the CFUs were recorded. Nontreated control systems received no drug treatment.

In the second HFS-TB study, the two-drug combination of tedizolid [standard dose] plus high dose moxifloxacin [800 mg/daily] was tested for sterilizing activity over 42 days, two replicate HFS-TB units per treatment regimen. Twenty mL of the SRB culture was inoculated into the peripheral compartment of each of the HFS-TB units. Sampling of the central compartment was performed as described above to validate the concentration-time profile of tedizolid and moxifloxacin. The peripheral compartments were sampled on days 0, 3, 7, 14, 21, 28, 35, and 42 and samples were processed to determine the total and drug resistant bacterial burden as described above, as well as TTP by inoculating the MGIT tubes.

In the third set of HFS-TB experiment, the *M. tuberculosis* SS18b strain was transformed to the NRP state by culturing in media without streptomycin supplementation (Zhang et al., 2012) and treated with either three-drug experimental combination of tedizolid, moxifloxacin, and faropenem or the standard regimen consisting of isoniazid plus rifampin plus pyrazinamide. There were three replicate HFS-TB units per treatment regimen. The half-lives of the drugs mimicked in the HFS-TB were 12 h for tedizolid, moxifloxacin, and pyrazinamide; 3 h for isoniazid and rifampin; and 1 h for faropenem (Srivastava et al., 2011a; Zhang et al., 2012; Deshpande et al., 2016a). Since the experiments were carried out in acidic environment using acidified media to a pH of 5.8, this set of experiments also served as a validation of the sterilizing activity in addition to determination of the killing efficacy of this combination against the NRP subpopulation of *M. tuberculosis*. Twenty mL of the NRP cultures were inoculated into the peripheral compartment of each of the HFS-TB units. The treatment regimens were as follows: standard dose of tedizolid once daily plus 800 mg of moxifloxacin daily plus faropenem twice daily to achieve 66% time-above-MIC (%T_MIC_) (Deshpande et al., 2016a); three-drug combination of isoniazid 300 mg/day, rifampin 600 mg/day, and pyrazinamide 1.5 g/day; and nontreated controls. All HFS-TB except the nontreated controls were sampled for drug concentration measurements and enumeration of the bacterial burden and TTP as described above. Sampling of the central compartment to validate the concentration-time profile of the drugs and of the peripheral compartment on days 0, 3, 7, 14, 21, 28, 35, 42, 49, and 56 was performed to determine the total and drug resistant bacterial burden as described above. The MGIT tubes were also inoculated to record the TTP as indication of *Mtb* growth.

### Pharmacokinetics/Pharmacodynamics Analysis

We used previously published methods, without any change, to measure the drug concentrations in each of the HFS-TB samples (Srivastava et al., 2011a; Srivastava et al., 2011b; Deshpande et al., 2016a; Srivastava et al., 2018). ADAPT (D’Argenio and Schumitzky, 1997) was used for pharmacokinetic analysis with the modeling steps as described in the past, including the one-compartment model with first-order input and elimination (Gumbo et al., 2009; Srivastava et al., 2011a). We used two measures of bacterial burden: total *Mtb* log_10_ CFU/ml and TTP in days. The linear regression model [for the CFU/ml readouts] was used to calculate the kill slopes of the combination regimens and one-way analysis of variance (ANOVA) was used to compare the regimens where the CFU readouts and therapeutic regimens were used as the dependent and independent variable, respectively. All the statistical analysis was performed using GraphPad Prism v8 (La Jolla, CA, United States).

## Results

The drug concentration-profiles achieved in the HFS-TB in three different experiments are shown in Supplementary Figures 1A–E, and the regression between the observed vs. model predicted concentration is shown in Supplementary Figure 1F where an *r* ^2^ = 0.93 shows good model fit and minimal bias.

**FIGURE 1:**
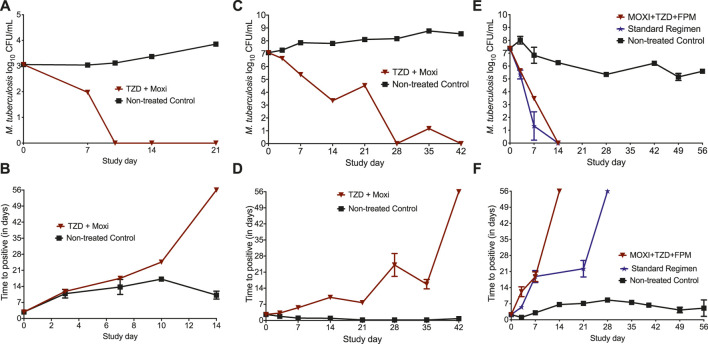
Changes in the bacterial burden in the HFS-TB treated with different treatment regimens. The data point is presented as mean ± standard error. In the bactericidal activity study, total microbial kill was recorded by both **(A)** CFU and **(B)** TTP measures in 14 and 10 days, respectively. In the HFS-TB experiment with starting bacterial burden of 7.07 log_10_ CFU/ml SRB population at acidic pH of 5.8, both **(C)** CFU and **(D)** TTP readouts were negative on day 42 indicating sterilization. **(E)** CFU and **(F)** TTP results of the HFS-TB experiment with *M. tuberculosis* SS18b, where both three-drug experimental regimen and the standard treatment regimen killed 7.35 log_10_ CFU/ml NRP population. *A decline in the NRP nontreated control is due to the fact that, in absence of streptomycin, *M. tuberculosis* SS18b strain stops growing and is due to the sample collected from HFS-TB units at each time point for bacterial culture.

The MIC of tedizolid and moxifloxacin against *Mtb* H37Rv was 0.25 mg/l, whereas the MIC of tedizolid, moxifloxacin, and faropenem against SS18b strain were 0.25, 0.5, and 4 mg/l, respectively. The changes in the bacterial burden over time, measured using TTP and CFU/ml, are shown in Figure 1. Figure 1A show the CFU/ml readouts in the HFS-TB where no bacterial growth was recorded on study day 10. The results of the MGIT-derived [TTP] readouts for the bactericidal activity of HFS-TB using the LPG cultures are shown in Figure 1B. The higher the bacterial burden in the HFS-TB, lower the TTP. After 14 days of treatment with tedizolid plus high-dose moxifloxacin combination, the MGIT show no growth units after 56 days of incubation, indicating total microbial kill in the drug-treated HFS-TB units. The day-21 TTP readouts could not be recorded due to a technical error; however, given that prior time point showed no growth units, it is assumed that there were no viable bacteria left in the systems.

The results of the sterilizing activity study, with *Mtb* cultures growing under acidic condition, are shown in Figures 1C,D. The reduction in total bacterial counts, as log_10_ CFU/ml, is shown in Figure 1C. No bacterial colony was recorded on day 28, followed by a transient regrowth on day 35. The TTP readouts (Figure 1C) showed that sterilization was achieved after 42 days of treatment with tedizolid plus high-dose moxifloxacin. Since MGIT is more sensitive compared to the solid agar-based culture method, we considered that sterilization of the HFS-TB units inoculated with the SRB *M. tuberculosis* was achieved after 42 days of treatment with tedizolid-moxifloxacin dual combination therapy.

In Figures 1E,F, we show the results of the three-drug combination used to treat the NRP *Mtb* SS18b. By the CFU measure (Figure 1E), both three-drug regimens killed the total NRP population in 14 days. As shown in Figure 1E, when TTP was used as a pharmacodynamics measure, the three-drug combination of tedizolid-moxifloxacin-faropenem showed negative culture after 14 days of therapy compared to the 21 days with the standard treatment regimen of isoniazid-rifampin-pyrazinamide. The kill rate with two- and three-drug regimens, calculated using the linear regression, is shown in Table 1. A negative growth rate in the NRP nontreated control (Table 1) is due to the fact that, in absence of streptomycin, *M. tuberculosis* SS18b strain stopped growing and a decline in total bacterial burden is due to the sample collected from HFS-TB units at each time point. Next, one-way ANOVA analysis was performed to compare the treatment regimens using the CFU readouts as dependent variable. The difference in the microbial kill between the standard and the experimental regimen was not significantly different [*p* = 0.671]. There were no drug resistance colonies recorded in the three HFS-TB experiments for tedizolid, moxifloxacin, and faropenem on agar supplemented with 3X MIC concentration.

**TABLE 1:** Growth rate of *M. tuberculosis* in nontreated HFS-TB and kill rate with experimental and standard regimen.

	TZD-MOXI	TZD-MOXI-FPM	Standard therapy	Nontreated control
**LPG**	−0.28 ± 0.12	Not tested	Not tested	0.04 ± 0.011
**SRB**	−0.17 ± 0.027	Not tested	Not tested	0.035 ± 0.01
**NRP**	Not tested	−0.53 ± 0.09	−0.59 ± 0.08	−0.039 ± 0.01

TZD, tedizolid; MOXI, moxifloxacin; FPM, faropenem; LPG, log-phase growth; SRB, slowly replicating bacilli at acidic pH of 5.8; NRP, nonreplicating persisters. The growth and kill rates are given as log_10_ CFU/mL/day.

## Discussion

Since people with latent tuberculosis could develop active TB at some point of life when the immune system gets compromised, this population essentially serve as a reservoir for TB. Therefore, finding drugs and combination that can effectively kill NRP *Mtb* is crucial (Barry et al., 2009). There are several major findings in the present study. First, using the human-like pharmacokinetics of the drugs, we show that tedizolid and moxifloxacin combination can effectively kill different metabolic populations of *Mtb*, in essence rejecting the special population hypothesis (Mitchison, 1979) that drugs work on selected *Mtb* metabolic states. Second, the three-drug combination of tedizolid-moxifloxacin-faropenem is effective against the NRP *Mtb*, as confirmed by killing of 7.37 log_10_ CFU/ml after 21 days of treatment [using the TTP readouts]. Thus, this three-drug combination could be used in the clinics. Since, tedizolid, moxifloxacin, and faropenem are active against both drug susceptible and drug resistant *Mtb*, this combination regimen has a potential to be pan-TB regimen. However, studies on drug resistant strains remain to be done, as well as clinical validation.

The third finding is the NRP killing efficacy of the isoniazid-rifampin-pyrazinamide. Previously it was shown that the extent of NRP kill with rifampin varies in a dose-dependent manner (Zhang et al., 2012; Iacobino et al., 2017), isoniazid has minimal activity (Srivastava et al., 2011b; Swaminathan et al., 2016), and pyrazinamide barely had any effect against *Mtb* SS18b or NRP state (Zhang et al., 2012). We found that the standard therapy killed 7.35 log_10_ CFU/ml NRP *Mtb* in 21 days, where the kill was likely driven by rifampin. This finding, while goes against the conventional wisdom (Mitchison, 1979) that none of the drugs in the standard regime kill NRP or dormant *Mtb*, likely explains why the currently recommended standard therapy successfully cures a large proportion of patients.

Our study has some limitation. First, the starting bacterial burden in the bactericidal activity HFS-TB experiment was lower than intended. Thus, the faster kill rate of LPG with tedizolid and moxifloxacin combination may not truly represent the scenario with higher bacterial load in the lung lesions. Second, in the nontreated HFS-TB, the bacteria in the log-phase growth experiment grew only 0.796 log_10_ CFU/ml in 21 days. We are unsure how this slow growth rate might have affected the outcome of the bactericidal activity of the drug combination. Third, we did not test emergence of drug resistance to isoniazid, rifampin, and pyrazinamide. However, since the total bacterial population was killed by the three-drug standard combination regimen, it is unlikely that there would be any acquired drug resistance to these drugs.

To conclude, the experimental combination regimen of tedizolid, moxifloxacin, and faropenem could effectively kill NRP population of *Mtb*, and currently recommended standard regimen has equally good efficacy against NRP in the HFS-TB model. The clinical validation of these findings remains to be determined.

## Data Availability Statement

The raw data supporting the conclusions of this article will be made available by the authors, without undue reservation following institutional policies.

## Author Contributions

SS and TG designed the study; SS, KC, and DD performed the experiments; SS wrote the first draft of the manuscript. All authors read and approved the final version of the manuscript.

## Funding

This work was supported by the Eunice Kennedy Shriver National Institute of Child Health and Human Development (NICHD; 1R01HD099756-02) to SS.

## Conflict of Interest

TG founded Praedicare Inc. and is on the Governing Board of Kiara Health.

The remaining authors declare that the research was conducted in the absence of any commercial or financial relationships that could be construed as a potential conflict of interest.
