# Supplementary figures and images for: Tedizolid, Faropenem, and Moxifloxacin Combination With Potential Activity Against Nonreplicating Mycobacterium tuberculosis
Source: Front Pharmacol. 2021 Jan 19;11:616294. doi: 10.3389/fphar.2020.616294 (PMC7851080; doi:10.3389/fphar.2020.616294)

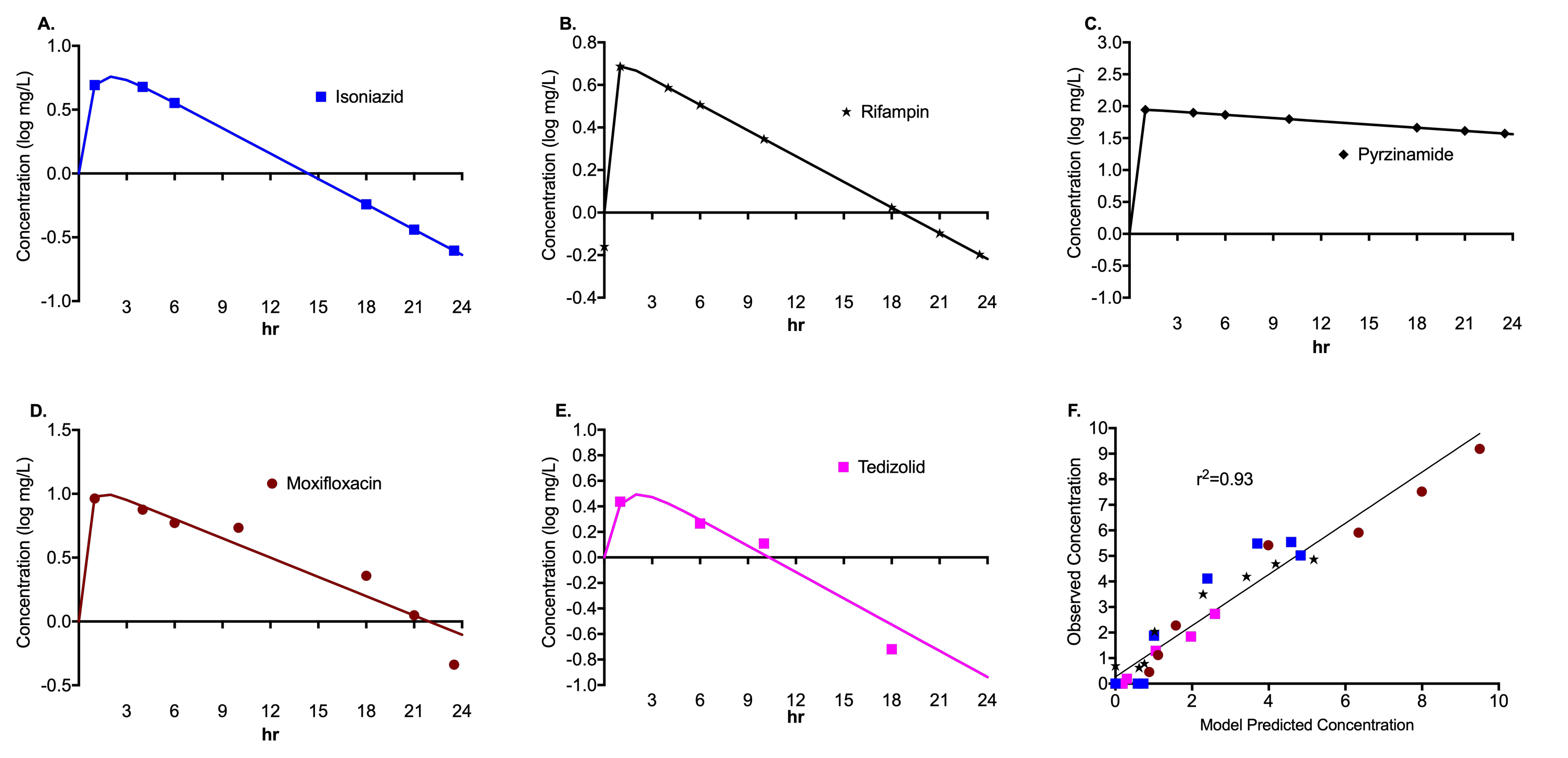

Supplement: Supplementary file 1 [file image1.tiff]
